# Sleep apnea syndrome comorbid with and without restless legs syndrome: differences in insomnia specific symptoms

**DOI:** 10.1007/s11325-020-02063-8

**Published:** 2020-04-25

**Authors:** Franziska Pistorius, Peter Geisler, Thomas C. Wetter, Tatjana Crönlein

**Affiliations:** Department of Psychiatry and Psychotherapy, University of Regensburg, Universitätsstraße 84, 93052 Regensburg, Germany

**Keywords:** Sleep apnea, Insomnia, Restless legs syndrome, Depression, Polysomnography

## Abstract

**Objective:**

Sleep Apnea Syndrome (SAS) is frequently comorbid with Restless Legs Syndrome (RLS). Both disorders are associated with disturbed sleep. However, data about insomnia specific symptoms in patients suffering from both sleep disorders (SAS-RLS) are rare.

**Methods:**

In a restrospective design, we investigated 202 patients suffering from SAS and SAS-RLS. All patients underwent polysomnography, performed a vigilance test (Quatember-Maly), and completed the Regensburg Insomnia Scale (RIS), Epworth Sleepiness Scale (ESS), Beck Depression Inventory-II (BDI-II), and a Morning Questionnaire (FZN). Differences in insomnia specific symptoms between SAS and SAS-RLS were calculated using ANOVA. In a secondary analysis, the differences in daytime sleepiness and depression were analyzed.

**Results:**

Of 202 patients, 42 (21%) had SAS-RLS. The proportion of women (60%) with SASRLS was higher than for men (40%) while men had had a higher proportion (71%) of SAS alone compared to women (29%), p < 0.0005. The RIS score was higher in SAS-RLS than in SAS. No differences were found in PSG data, ESS, BDI-II, or vigilance tests.

**Conclusions:**

Patients with both disorders SAS and RLS show a higher degree of insomnia-specific symptoms than for SAS alone and may profit from additional insomnia specific treatment.

## Introduction

Comorbidity between sleep disorders is often seen in daily clinical routine and is gaining more and more scientific attention in sleep research [1–5]. The prevalence of Restless Legs Syndrome (RLS) in patients with Sleep Apnea Syndrome varies between 7 and 36% [6, 7], which is higher than in normal population [7, 8]. It is known that Sleep Apnea Syndrome (SAS) as well as RLS are associated with sleep difficulties such as problems initiating and maintaining sleep, a short sleep time and impaired daytime functioning [1, 2, 9]. Moreover, SAS as well as RLS frequently seem to be associated with depression [10, 11]. RLS as well as SAS may be associated with insomnia specific emotional and cognitive symptoms of insomnia [12, 13]. However, there is a rare data about the question, whether patients who are suffering from both disorders (SAS-RLS) are more affected by psychological symptoms of insomnia disorder than patients just having SAS. The symptoms of insomnia disorder exceed disturbed sleep. These patients complain about impaired daytime performance and typically show sleep-related worries [14] that are recognized to fuel insomnia specific hyperarousal. The fact that SAS-RLS show more insomnia specific psychological symptoms may give further information regarding the etiology of insomnia as well as the need for possible additional treatment options such as Cognitive Behavior Therapy for Insomnia.

Rodrigues et al. compared 13 SAS and 17 SAS-RLS regarding sleep quality, polysomnographic data, daytime sleepiness, depression, and fatigue without finding any differences [15]. Another study comparing SAS with SAS-RLS showed that daytime sleepiness as well as difficulties initiating and maintaining sleep more than 3 times per week were more pronounced in SAS-RLS than in SAS [7].

In this study, we retrospectively investigated a large sample of inpatients suffering from SAS or SAS-RLS. All patients were studied regarding insomnia specific symptoms including severity, objective sleep data and subjective sleep parameters, and daytime fitness. We also assessed depression scores since it is known that insomnia patients show some overlap with depressive syndrome [16]. Furthermore, impaired daytime performance as a consequence of sleep loss is an issue in every sleep disorder. For patients suffering from insomnia, data is controversial suggesting a hyperarousal for compensating tiredness [17]. It was therefore interesting to investigate performance in psychomotoric tests in our samples.

## Materials and methods

A single-center retrospective inpatient study using charts and polysomnographic data was performed.

## Patients

All patients were diagnosed and treated in the sleep laboratory of Sleep Disorder Center Regensburg, Germany, in 2015 and 2016 in an inpatient setting.

Every patient had had a face-to-face interview and a physical examination in our sleep-clinic by a sleep specialist. Inclusion criteria were defined as (American Academy of Sleep Medicine: International Classification of Sleep Disorders: Diagnostic and Coding anual, 2005) AHI ≥ 5/h combined with cardinal symptoms of SAS or AHI ≥ 15/h. Patients with predominant obstructive events were included. RLS criteria (IRLSSG in 2002) were as follows: (1) urge to move the legs, usually accompanied by discomfort in the lower limbs (2) occurrence or worsening of symptoms in situations of rest or inactivity (e.g., in bed) (3) partially or totally relief of symptoms through movement, and (4) symptoms are worse or solely in the evening. RLS symptoms including frequency and severity were explored during the face-to-face interview to avoid false-positive RLS diagnoses. Exclusion criteria were the presence of severe psychiatric disorders such as a psychosis, cognitive disorder, alcohol abuse, or difficulties in German language and Parkinson disease. Patients who received RLS treatment or were treated with CPAP at the time of admission were also excluded.

All the untreated patients with SAS who were admitted for the first time to our sleep laboratory between 2015 and 2016 were screened for analysis. Fourteen patients were excluded because of following reasons: psychosis (*N* = 5), cognitive disorder (*N* = 4), difficulties in German language (*N* = 3), alcohol abuse (*N* = 1), Parkinson disease (N = 1), or incomplete data (*N* = 20).

## Measurements

### Polysomnography

A full cardio-respiratory polysomnographic recording night was performed and scored by sleep specialists according to the manual of the American Academy of Sleep Medicine. A polysomnography was performed in a separated bedroom in our sleep laboratory located in the clinic. During baseline nights, our usual recording times were conducted (10 pm to 6 am). The polysomnography included the electroencephalogram (frontal, central, and occipital leads, referenced to the contralateral mastoid), electrooculogram (alternative derivation, E_1_-F_pz_ and E_2_-F_pz_), electromyogram of the chin muscle, tibialis anterior muscles bilaterally, nasal airflow (pressure transducer) thoracic and abdominal respiration (uncalibrated induction plethysmography), oxygen saturation, electrocardiogram, and body position. Hypopnea definition B (alternative) was used. Sleep stages, periodic leg movements, and respiratory events were classified by a trained staff member according to the AASM manual. Respiratory event-associated movements were not classified in a separate category.

The following sleep parameters were analyzed: sleep onset latency (SOL); total sleep time (TST); wake time after sleep onset (WASO); sleep efficiency (SE); and NREM1, NREM 2, NREM 3, and REM sleep stages in % of sleep period time (SPT).

### Psychological tests

Regensburg Insomnia Scale (RIS) is a ten-item self-report inventory to evaluate the psychophysiological symptoms of insomnia, 0–40 scores, cut-off 12 [18]. Epworth Sleepiness Scale (ESS) is an eight-item questionnaire for measuring daytime sleepiness in adults, 0–24 scores, cut-off 10. Beck Depressions Inventory-II (BDI-II) 0–63 score, cut-off 12. In the morning after polysomnography, a Morning Questionnaire was given asking for sleep duration (in minutes), time to fall asleep (in minutes), and number of awakenings (unpublished). A sustained attention test (VIGIL, S1, Quatember and Maly) of the Vienna Test System (Version 5.10, Schuhfried GmbH, Mödling, Austria) was performed in all subjects.

### Analysis

For the statistical analysis, SPSS 24.0.0.1 (SPSS Inc., Chicago, IL, USA) was used. The two-tailed significance level was defined as *p* < .05.

Regarding age and BMI, SAS and SAS-RLS were compared by using student’s *t* tests. Differences in sex were calculated by using Chi^2^ test. In case of significant differences, age, gender, or BMI were included as cofactors in further comparisons. For calculating the differences in scores in ESS, BDI, RIS, parameters of Quatember-Maly, and sleep parameters, ANOVA was used. Missing data in ESS, BDI, RIS, or vigilance test were replaced by mean substitution (SPSS).

## Results

### Sample

The sample consisted of 202 patients showing sleep apnea (mean age of 55 years, SD = 11.0) with 71 (35%) women. Mean BMI was 31 kg/m^2^ (SD = 6). Forty-two patients (21%) had comorbid RLS.

### Clinical differences between SAS-RLS and SAS

In SAS-RLS, the number of women (60%) was significantly higher (*p* < .0005) than in SAS (29%). Gender was used as covariate in further calculations. No differences in age or BMI were found (see Table 1).

**Table 1:** Epidemiological data of SAS-RLS and SAS

	160 SAS	42 SAS-RLS	*p* value
Age	54 (σ = 11.4)	57 (σ = 8.8)	.145 (*t* test)
Sex	♀46 (29%),♂114 (71%)	♀25 (60%),♂17 (40%)	< .0005 (Chi^2^)
BMI	31 (σ = 5.4, *N* = 153)	32 (σ = 8, *N* = 40)	.301 (*t* test)

### Insomnia specific differences between SAS-RLS and RLS

The RIS scores in SAS-RLS were significantly higher (*p* = .005). Significant differences were found in the items “I wake up too early”, “I wake up from the slightest sound”, “I feel that I have not slept all night”, “I think a lot about my sleep”, and “I am afraid to go to bed because of my disturbed sleep” (see Table 2).

**Table 2:** Differences between 160 SAS and 42 SAS-RLS in insomnia symptoms measured by Regensburg Insomnia Scale (RIS)

	160 SAS	42 SAS-RLS	ANOVA	
RIS items	Mean and SD	Mean and SD	Factor group	Factor gender
RIS score (*N* = 198)	15.9 (σ = 7.88)	19.6 (σ = 1.14)	*F* = 7.931; *p* = .005	*F* = 5.077; *p* = .025
How many minutes do you need to fall asleep?	0.88 (σ = 1.14)	1.02 (σ = 1.24)	*F* = .471; *p* = .493	*F* = .154; *p* = .696
How many hours do you sleep during the night?	1.03 (σ = 0.92)	1.19 (σ = 0.99)	*F* = 1.001; *p* = .318	*F* = .760; *p* = .385
My sleep is disturbed	2.25 (σ = 1.28)	2.52 (σ = 1.4)	*F* = 1.457; *p* = .229	*F* = .286; *p* = .594
I wake up too early	2.12 (σ = 1.24)	2.58 (σ = 1.31)	*F* = 4.461; *p* = .036	*F* = 4.581; *p* = .034
I wake up from the slightest sound	1.69 (σ = 1.34)	2.5 (σ = 1.27)	*F* = 12.295; *p* = .001	*F* = 9.614; *p* = .002
I feel that I have not slept all night	1.88 (σ =2.02)	2.36 (σ = 1.25)	*F* = 8.117; *p* = .005	*F* = 6.019; *p* = .015
I think a lot about my sleep	1.81 (σ = 1.28)	2.31 (σ = 1.28)	*F* = 4.956; *p* = .027	*F* = 4.529; *p* = .035
I am afraid to go to bed because of my disturbed sleep	1.17 (σ = 1.33)	1.81 (σ = 1.4)	*F* = 7.405; *p* = .007	*F* = 5.895; *p* = .016
I feel fit during the day	2.23 (σ = 1.22)	2.48 (σ = 1.33)	*F* = 1.284; *p* = .258	*F* = 1.191; *p* = .277
I take sleeping pills in order to get to sleep	0.8 (σ = 1.41)	0.88 (σ = 1.5)	*F* = .12; *p* = .73	*F* = .115; *p* = .734

### Differences in sleep parameters between SAS and SAS-RLS

SAS-RLS and SAS did not differ in any PSG parameters, except for SAS-RLS showing a tendency of higher arousals indices (*p* = .056). There was a significant difference seen in PLM-Index (*p* = .04) and PLM-Arousal-Index (*p* < .0005) between groups (see Table 3).

**Table 3:** Polysomnographic data of 160 SAS patients and 42 SAS-RLS patients

	160 SAS	42 SAS-RLS		
	Mean and SD	Mean and SD	Factor group	Factor Gender
Time in bed (TIB)	452 min (σ = 23.68)	451 min (σ = 34.2)	*F* = .878; *p* = .878	*F* = .014; *p* = .907
Sleep period time (SPT)	420 min (σ = 41.82)	431 min (σ = 44.84)	*F* = 2.167; *p* = .143	*F* = 2.7; *p* = .102
Total sleep time (TST)	338 min (σ = 70.95)	336 min (σ = 68.16)	*F* = .014; p = .907	*F* = .022; *p* = .881
Wake time after sleep onset (WASO)	85 min (σ = 55.41)	95 min (σ = 51.4)	*F* = 1.267; *p* = .262	*F* = 1.674; *p* = .197
Sleep latency (SOL)	25 min (σ = 31.82)	17 min (σ = 20.08)	*F* = 2.486; *p* = .116	*F* = 4.379; *p* = .038
Sleep efficiency (SE)	74% (σ = 16.08)	74% (σ = 13.03)	*F* = .007; *p* = .933	*F* = .000; *p* = .990
N1 (% of SPT)	16% (σ = 9.05)	16% (σ = 8.94)	*F* = .150; *p* = .699	*F* = .022; *p* = .883
N2 (% of SPT)	37% (σ = 11.02)	35% (σ = 11.01)	*F* = 1.092; *p* = .297	*F* = .696; *p* = .405
N3 (% of SPT)	15% (σ = 9.69)	16% (σ = 10.53)	*F* = .168; *p* = .682	*F* = .282; *p* = .596
REM (% of SPT)	11% (σ = 6.01)	11% (σ = 6.16)	*F* = .013; *p* = .910	*F* = .033; *p* = .857
Arousal index (AI)	34/h (σ = 19.52)	41/h (σ = 20.03)	*F = 3.682; p = .056*	*F* = 4.926; *p* = 028
PLM index	31/h (σ = 27.35)	41/h (σ = 35.45)	*F* = 4.272; *p* = .040	*F* = 4.007; *p* = .047
PLM-Arousal index	6/h (σ = 7.25)	14/h (σ = 15,03)	*F* = 24.473; *p* < .0005	*F* = 20.669; p < .0005
Apnea-Hypopnea index (AHI)	27/h (σ = 28,14)	27/h (σ = 19.71)	*F* = .000; *p* = .991	*F* = .159; *p* = .690
Apnea index	15/h (σ = 23.71)	9/h (σ = 12.18)	*F* = 2.733; *p* = .100	*F* = 1.195; *p* = .276
Desaturation index	20/h (σ = 21.47)	19% (σ = 20.04)	*F* = .128; *p* = .721	*F* = .013; *p* = .908
Mean saturation	93% (σ = 1.89)	92% (σ = 1.82)	*F* = .784; *p* = .377	*F* = .238; *p* = .627
Arousal-breathing index	17/h (σ = 18.96)	18/h (σ = 14.04)	*F* = .124; *p* = .726	*F* = .682; *p* = .410

The analysis of the scores of ESS, BD-III, Morning questionnaire, and parameters of sustained attention test did not show any differences between SAS and SAS-RLS (see Tables 4 and 5).

**Table 4:** Epworth Sleepiness Scale and Beck Depressions Inventory-II of SAS and SAS-RLS

	SAS	SAS-RLS	Factor group	Factor gender
ESS	9.5 (σ = 5,09)	11.0 (σ = 4,55)	*F* = 2.918; *p* = .089	*F* = 2.616; *p* = .107
Sitting and reading	1.2 (σ = 0,96)	1.67 (σ = 0,93)	*F* = 7.828; *p* = .006	*F* = 7.105; *p* = .008
Watching TV	2.01 (σ = 0,98)	2.25 (σ = 0,8)	*F* = 1.953; *p* = .164	*F* = 1.407; *p* = .237
Sitting, inactive in a public place	1.32 (σ = 1,03)	1.37 (σ = 1,07)	*F* = .093; *p* = .761	*F* = .033; *p* = .857
As a passenger in a car for an hour without a break	1.11 (σ = 1,04)	1.41 (σ = 0,97)	*F* = 2.916; *p* = .089	*F* = 1.705; *p* = .193
Lying down to rest in the afternoon when circumstances permit	2.13 (σ = 0,99)	2.15 (σ = 1,01)	*F* = .015; *p* = .903	*F* = .148; *p* = .701
Sitting and talking to someone	0.33 (σ = 0,5)	0.52 (σ = 0,78)	*F* = 3.581; *p* = .060	*F* = 4.296; *p* = .040
Sitting quietly after a lunch without alcohol	1.08 (σ = 0,99)	1.27 (σ = 1,03)	*F* = 1.158; *p* = .283	*F* = .768; *p* = .382
In a car while stopping for a few minutes in the traffic	0.31 (σ = 0.6)	0.34 (σ = 0.66)	*F* = .102; *p* = .750	*F* = .554; *p* = .458
**BDI**	12.8 (σ = 9.77)	15.9 (σ = 10.63)	*F* = 2.883; *p* = .091	*F* = 1.67; *p* = .198
A-Feeling sad	0.43 (σ = 0.72)	0.32 (σ = 0.63)	*F* = .68; *p* = .411	*F* = 1.166; *p* = .282
B-Discouraged about the future	0.45 (σ = 0.85)	0.58 (σ = 0.89)	*F* = .638; *p* = .426	*F* = .449; *p* = .504
C-Failure	0.41 (σ = 0.77)	0.59 (σ = 0.83)	*F* = 1.589; *p* = .209	*F* = .467; *p* = .495
D-Satisfaction	0.81 (σ = 0.74)	0.85 (σ = 0.74)	*F* = .097; *p* = .755	*F* = .217; *p* = .642
E-Guiltiness	0.45 (σ = 0.76)	0.51 (σ = 0.65)	*F* = .210; *p* = .647	*F* = .000; *p* = .994
F-Feel being punished	0.55 (σ = 1.03)	0.78 (σ = 1.16)	*F* = 1.44; *p* = .232	*F* = 1.859; *p* = .174
G-Disappointment	0.38 (σ = 0.66)	0.57 (σ = 0.77)	*F* = 2.293; *p* = .132	*F* = 1.145; *p* = .286
H-Self-concept	0.53 (σ = 0.69)	0.68 (σ = 0.85)	*F* = 1.226; *p* = .27	*F* = .341; *p* = .56
I-Suicidal thoughts	0.24 (σ = 0.44)	0.35 (σ = 0.43)	*F* = .021; *p* = .885	*F* = .192; *p* = .662
J-Crying	0.34 (σ = 0.76)	0.65 (σ = 1.06)	*F* = 4.121; *p* = .044	*F* = 2.728; *p* = .10
K-Irritation	0.83 (σ = 0.84)	0.86 (σ = 0.89)	*F* = .041; *p* = .84	*F* = .197; *p* = .658
L-Interest in other people	0.51 (σ = 0.77)	0.7 (σ = 0.85)	*F* = 1.772; *p* = .185	*F* = 1.795; *p* = .182
M-Come to a decision	0.93 (σ = 0.84)	0.95 (σ = 0.82)	*F* = .011; *p* = .916	*F* = .061; *p* = .805
N-Feel looking un−/attractive	0.5 (σ = 0.86)	0.86 (σ = 1.13)	*F* = 4.595; *p* = .033	*F* = 1.587; *p* = .209
O-Able to do work	1.01 (σ = 0.82)	1.08 (σ = 0.72)	*F* = .249; *p* = .619	*F* = .167; *p* = .683
P-Sleep	1.23 (σ = 0.92)	1.68 (σ = 0.92)	*F* = 6.894; *p* = .009	*F* = 4.943; *p* = .027
Q-Getting tired	1.13 (σ = 0.82)	1.38 (σ = 0.72)	*F* = 3.077; *p* = .081	*F* = 2.59; *p* = .109
R-Appetite	0.26 (σ = 0.6)	0.22 (σ = 0.42)	*F* = .151; *p* = .698	*F* = .103; *p* = .749
S-Losing weight	0.33 (σ = 0.77)	0.23 (σ = 0.63)	*F* = .491; *p* = .485	*F* = .685; *p* = .409
T-Worrying about health	0.64 (σ = 0.72)	0.8 (σ = 0.62)	*F* = 1.614; *p* = .206	*F* = 1.97; *p* = .162
U-Interest in sex	0.85 (σ = 1.03)	1.35 (σ = 1.27)	*F* = 6.346; *p* = .013	*F* = 1.67; *p* = .198

**Table 5:** Sustained attention test of SAS and SAS-RLS

	SAS	SAS-RLS	Factor group	Factor Gender
Mean reaction time (sec.)	0.51 s (σ = 0.11)	0.54 s (σ = 0.11)	*F* = 1.468; *p* = .227	*F* = .185; *p* = .667
Number of omissions	4.77 (σ = 8.89)	6.05 (σ = 14.2)	*F* = .485; *p* = .487	*F* = .087; *p* = .768
False-positive reactions	3.59 (σ = 9.7)	2.74 (σ = 3.41)	*F* = .286; *p* = .593	*F* = .654; *p* = .42

## Discussion

The prevalence of 21% of sleep apnea patients suffering from RLS in our sample is comparable to the data published in ISAC study [1, 7], and it is higher than the prevalence of RLS found in normal population (4 to 14%) [8, 19]. The higher proportion of women in our sample of SAS-RLS could be explained by the high prevalence of women in RLS in general population [8, 20]. However, there are also studies finding no difference between women and men regarding RLS prevalence [21]. Further studies should clarify this issue.

Our main result is that SAS-RLS complained more psychological insomnia specific symptoms than SAS. It has been shown before that RLS patients may show insomnia specific characteristics such as sleep disruptive habits and cognitions [12], similar data was found in patients suffering from sleep apnea [13]. To our knowledge, this is the first study showing a higher incidence of insomnia specific symptoms such as sleep related worries in patients suffering from both. The question whether disturbed sleep quality is caused by RLS, SAS or insomnia disorder cannot be cleared in this study and should be clarified by further research. However, the fact that insomnia comorbid with other sleep disorders should be treated with insomnia-specific methods is an important feature of insomnia management [22]. Therefore a combination of SAS and RLS therapy with elements of Cognitive Behavior Therapy for Insomnia such as psycho-education could be beneficial.

Findings in the PSG data of SAS and SAS-RLS are comparable to previous data showing no differences regarding TST, SE, WASO, stages of sleep, or breathing parameters [15]. However, since SAS-RLS show more insomnia specific symptoms, it should have been expected that they display more difficulties falling asleep in our sleep laboratory night. This was not the case and probably due to the sleep lab situation and the fact that we just had one diagnostic night. Further studies with more successive nights would be useful to clarify this topic. the sleep structure of both SAS and SAS-RLS tended to be low, but to a similar extent. Arousal Index, PLM Index, and PLM-Arousal Index were more pronounced in SAS-RLS. This is in line with earlier data showing that the RLS in SAS patients was an independent risk factor for difficulties maintaining sleep [1]. Our results showing that SAS-RLS patients suffer more from light sleep and early awakening as had been measured with the RIS may reflect this data.

However, the negative impact of periodic limb movements in sleep on sleep quality has been discussed controversially [23, 24]. The fact that we did not address respiratory event associated movements [25] can be seen as a limiting factor of our study. These events are known to be more frequent in patients showing sleep apnea comorbid with periodic leg movements in sleep. It is hypothesized that they indicate a more severe form of breathing disorder. A recently published study shows that CPAP therapy enhances PLM indices in SAS patients [26]. Further studies are necessary to address this complex phenomenon. In our study, we found a trend toward more arousals in SAS-RLS.

A secondary result is the fact that there is no difference in the degree of depression between SAS and SAS-RLS. Both SAS and SAS-RLS had mean BDI-II score of 13 and 16 indicating mild depressiveness. Depressive symptoms in SAS or RLS had been shown before [27, 28]. Moreover, SAS and SAS-RLS did not differ in psychomotoric performance, both showing impaired performance.

## Conclusion

RLS is a frequent comorbid disorder in patients suffering from SAS. SAS-RLS show a higher degree in insomnia specific psychological symptoms and may benefit from the elements of insomnia specific Cognitive behavior therapy.
